# LED Light Intensity Regulates Nitrogen Assimilation Enzyme Activity and Metabolic Responses in Iceberg and Leaf Lettuce (*Lactuca sativa* L.)

**DOI:** 10.3390/plants15091321

**Published:** 2026-04-25

**Authors:** Nga T. T. Nguyen, Nasratullah Habibi, Naveedullah Sediqui, Oliveira Leonardo de Almeida, Maryam Dabirimirhosseinloo, Naoki Terada, Atsushi Sanada, Kaihei Koshio

**Affiliations:** 1 Faculty of International Agriculture and Food Studies, Tokyo University of Agriculture, Sakuragaoka 1-1-1, Setagaya-ku, Tokyo 156-8502, Japan; nasratullah.habibi14@gmail.com (N.H.); naweedone@gmail.com (N.S.); leonardoaaloliveira@gmail.com (O.L.d.A.); 13423403@nodai.ac.jp (M.D.); nt204361@nodai.ac.jp (N.T.); a3sanada@nodai.ac.jp (A.S.); koshio@nodai.ac.jp (K.K.); 2 Faculty of Food Science and Technology, Vietnam National University of Agriculture, Gia Lam, Hanoi 12400, Vietnam; 3 Faculty of Agriculture, Balkh University, Balkh 1701, Afghanistan; 4 Department of Horticulture, Faculty of Agriculture, Alberoni University, Kohistan 1254, Afghanistan

**Keywords:** lettuce, light intensity, nitrogen metabolism, nitrate accumulation, metabolomics, ascorbic acid, cultivar-different responses, controlled-environment agriculture

## Abstract

Light availability is a key environmental factor regulating nitrogen assimilation, carbon metabolism, and nutritional quality in leafy vegetables grown in controlled environments. However, how practical lighting regimes used in plant factories with artificial lighting (PFALs) influence the coordination between nitrogen assimilation and central carbon metabolism across different lettuce cultivar types remains insufficiently understood. This study investigated how moderate differences in photosynthetic photon flux density (PPFD) influence nitrogen metabolism and metabolic coordination in hydroponically cultivated lettuce. Two cultivars representing contrasting morphological types, iceberg lettuce (‘Celebration’) and leaf lettuce (‘Sunny’), were grown under LED light intensities of 150 and 200 µmol·m^−2^·s^−1^. Nitrate, nitrite, and ammonium concentrations were measured together with the activities of nitrate reductase (NRA) and nitrite reductase (NiRA), as well as ascorbic acid content. Metabolomic profiling was additionally performed to characterize broader metabolic responses. Higher light intensity enhanced nitrate reduction capacity in both cultivars, but the resulting patterns of nitrogen accumulation were strongly genotype-dependent. The leaf lettuce cultivar ‘Sunny’ exhibited increased NRA and reduced nitrate accumulation under higher light intensity, whereas the iceberg lettuce cultivar ‘Celebration’ accumulated more nitrate under the same conditions. Ammonium responses further suggested differences in downstream nitrogen assimilation processes. Elevated light intensity also increased ascorbic acid levels in both cultivars. Metabolomic analysis revealed contrasting cultivar-specific shifts in central carbon metabolism, particularly involving soluble sugars and tricarboxylic acid cycle intermediates, indicating differential coordination between carbon metabolism and nitrogen utilization. Overall, these findings demonstrate that moderate changes in light intensity within the practical PFAL cultivation range can significantly influence the integration of carbon and nitrogen metabolism in lettuce. Importantly, cultivar-specific physiological traits determine how these metabolic responses translate into nitrate accumulation and nutritional quality in controlled-environment production systems.

## 1. Introduction

Lettuce (*Lactuca sativa* L.) is one of the most widely cultivated leafy vegetables worldwide and represents an important crop in both conventional agriculture and controlled-environment production systems. Belonging to the family Asteraceae, lettuce exhibits considerable morphological diversity and includes several major horticultural types such as (1) iceberg lettuce (also known as crisphead lettuce), (2) butterhead lettuce, (3) romaine or cos lettuce, (4) leaf lettuce, (5) stem or stalk (Asparagus) lettuce, (6) Latin lettuce [1,2]. Owing to its relatively short growth cycle, high nutritional value, and suitability for hydroponic cultivation, lettuce has become a model crop for intensive indoor production systems such as PFAL with high nutritive value [3,4,5] and medicinal importance [6,7,8,9,10,11].

Despite its nutritional benefits, lettuce is also known for its tendency to accumulate relatively high levels of nitrate in leaf tissues. In some cases, nitrate concentrations in fresh lettuce can exceed 3500 mg/kg fresh weight [12]. Nitrate (NO_3_^−^), recognized as a crucial nitrogen source for the growth and development of plants, is extensively utilized in vegetable cultivation, particularly within hydroponic systems. Lettuce serves as a hyperaccumulator of NO_3_^−^ and demonstrates a remarkable capacity to accumulate this compound in its leaves [13]. NO_3_^−^ exists as a non-toxic form of nitrogen for plants; however, when it is converted into nitrite (NO_2_^−^), it poses potential hazards to humans and animals. Elevated NO_3_^−^ concentrations can result in correspondingly high NO_2_^−^ levels [14,15]. NO_2_^−^ is extremely toxic and can diminish the oxygen supply in the human body, potentially leading to conditions such as blue baby syndrome [16]. Additionally, NO_2_^−^ plays a role in the formation of nitrosamines, which are associated with cancer risk [17,18]. Consequently, regulatory limits have been established for nitrate concentrations in leafy vegetables. For example, the European Commission has established a maximum permissible limit for NO_3_^−^ concentration in lettuce grown under protected cultivation at 2500 mg NO_3_^−^/kg of fresh weight (FW) for “Iceberg” type and 4000–5000 mg NO_3_^−^/kg FW for the other fresh lettuce [19]. Reducing NO_3_^−^ accumulation in lettuce therefore remains an important objective for improving both food safety and crop quality.

**Figure 1:**
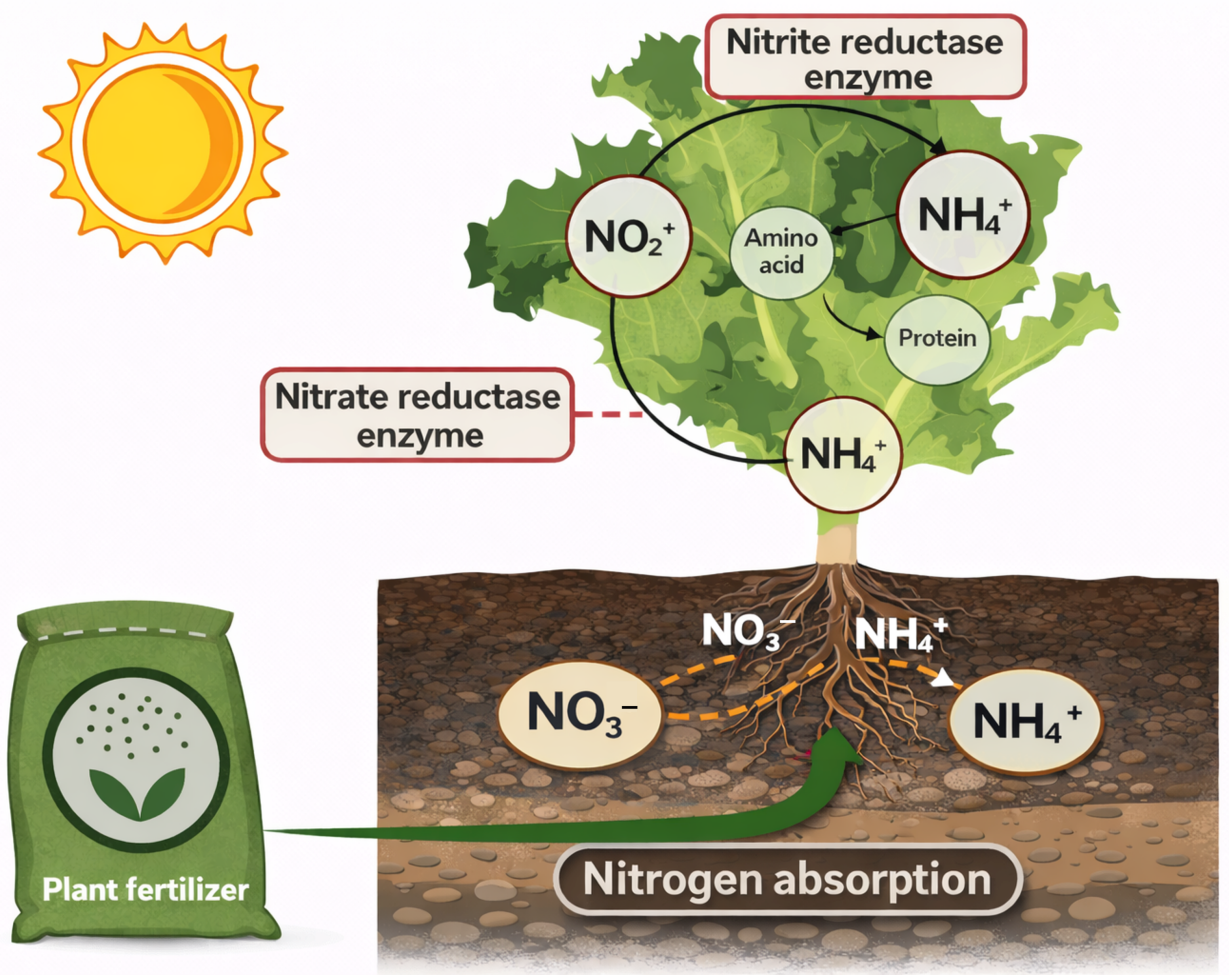
Nitrogen conversion and metabolic integration in lettuce.

Nitrogen assimilation in lettuce involves a coordinated sequence of biochemical reactions linking nitrate reduction with carbon metabolism (Figure 1). Lettuce has the capability to absorb nitrogen in the forms of NO_3_^−^ or ammonium (NH_4_^+^) from fertilizers through its roots [20]. Within the leaves, the enzyme nitrate reductase (NR) facilitates the biochemical reduction of NO_3_^−^ to NO_2_^−^, while nitrite reductase (NiR) is responsible for converting NO_2_^−^ to NH_4_^+^ [21,22]. The resulting NH_4_^+^ is then incorporated into amino acids through downstream assimilation pathways. Because NO_3_^−^ reduction requires both reducing power and carbon skeletons, nitrogen assimilation is closely coupled with photosynthetic carbon metabolism and the tricarboxylic acid (TCA) cycle.

Figure 1 illustrates a conceptual framework of nitrogen conversion and its metabolic integration in lettuce leaves. Nitrate absorbed by roots is transported to leaf tissues, where it is reduced sequentially by NR and NiR enzymes. The resulting ammonium is incorporated into amino acids using carbon skeletons derived from primary carbon metabolism, including sugars produced through photosynthesis and organic acids from the TCA cycle. Consequently, the efficiency of NO_3_^−^ assimilation depends not only on nitrogen supply [23,24] but also on environmental factors such as light intensity, as described below.

Light availability plays a central role in regulating these processes. Previous studies have demonstrated that increasing photosynthetic photon flux density (PPFD) can enhance lettuce growth, biomass accumulation, and nitrate assimilation capacity. For example, Gaudreau et al. reported that supplemental lighting significantly increased lettuce biomass and head firmness compared with plants grown under natural light conditions [25].

Other studies have shown that lettuce dry mass and leaf number increase with increasing PPFD up to 200 µmol·m^−2^·s^−1^ and this light intensity is optimal for the production of lettuce plug transplants [26]. However, excessively high light intensities may induce oxidative stress or physiological disorders such as tip burn, particularly in head-forming lettuce cultivars [27].

In modern indoor agriculture, hydroponic lettuce is produced in large quantities in PFALs, particularly in Japan [28]. Within these systems, light intensity must be carefully optimized to balance plant growth, energy consumption, and crop quality. Many studies report that lettuce grown under controlled lighting conditions shows an optimal physiological performance within a PPFD range of 150–250 µmol·m^−2^·s^−1^, which has therefore become a commonly adopted operational range in PFAL cultivation systems [26,27,29,30]. Intensities below 150 µmol·m^−2^·s^−1^ may restrict growth and biomass accumulation in leaf lettuce [29], whereas higher irradiance (200–300 µmol·m^−2^·s^−1^) may increase the incidence of tipburn in heading cultivars due to rapid growth and calcium imbalance in internal tissues [31]. Furthermore, semi-heading and heading cultivars exhibit tipburn at elevated light intensities above 240 µmol·m^−2^·s^−1^ in indoor horticulture systems, reinforcing the need for moderated PPFD to balance growth and physiological stability [32].

Importantly, lettuce cultivars differ markedly in their morphological structure and physiological responses to environmental conditions. Leaf lettuce typically forms an open canopy architecture that allows relatively uniform light penetration across leaves. In contrast, iceberg (crisphead) lettuce develops a compact head composed of multiple tightly overlapping leaves. This dense structure can limit internal light penetration within the canopy, potentially influencing photosynthetic activity, nitrogen assimilation efficiency, and metabolite accumulation in inner leaf tissues.

Although numerous studies have explored the effects of light intensity on lettuce growth and productivity, less attention has been given to how practical PFAL light regimes influence the integration of nitrogen assimilation, carbon metabolism, and metabolite profiles across different lettuce cultivar types. Most previous studies have focused primarily on nitrate concentration alone, without examining the broader metabolic coordination linking nitrogen assimilation with carbon metabolism and bioactive compounds.

Therefore, the present study aimed to investigate how moderate differences in light intensity within a practical PFAL cultivation range influence nitrogen metabolism, carbon metabolic pathways, and bioactive metabolite accumulation in lettuce. Two lettuce cultivars with contrasting morphologies were used, the iceberg lettuce cultivar ‘Celebration’ and the leaf lettuce cultivar ‘Sunny’. Therefore, two light intensities, 150 µmol·m^−2^·s^−1^ and 200 µmol·m^−2^·s^−1^, were chosen to establish a comparative baseline that balances growth with the prevention of physiological necrosis for the studied lettuce. Specifically, we evaluated inorganic nitrogen pools (NO_3_^−^, NO_2_^−^, and NH_4_^+^), the NRA and NiRA; primary carbon metabolites including glucose, sucrose, and TCA cycle intermediates; and several stress-related metabolites such as γ-aminobutyric acid (GABA), polyamines, and amino acids.

### Study Novelty and Significance

This study provides a comprehensive physiological and metabolomic assessment of lettuce responses to light intensity within the practical operational range commonly used in plant factories with artificial lighting. Unlike previous studies that primarily examined nitrate accumulation alone, the present work integrates inorganic nitrogen pools, nitrate assimilation enzymes, central carbon metabolites, and stress-related metabolites to elucidate the metabolic coordination underlying nitrate accumulation. Furthermore, by directly comparing leaf and crisphead lettuce cultivars with contrasting canopy architectures, this study reveals how structural differences between cultivars influence nitrogen assimilation efficiency under identical lighting regimes. These findings provide new insights into cultivar-specific metabolic strategies and offer practical guidance for optimizing lighting management and cultivar selection in controlled-environment lettuce production systems.

## 2. Results

### 2.1. Effects of Light Intensity on Iceberg Lettuce

**Figure 2:**
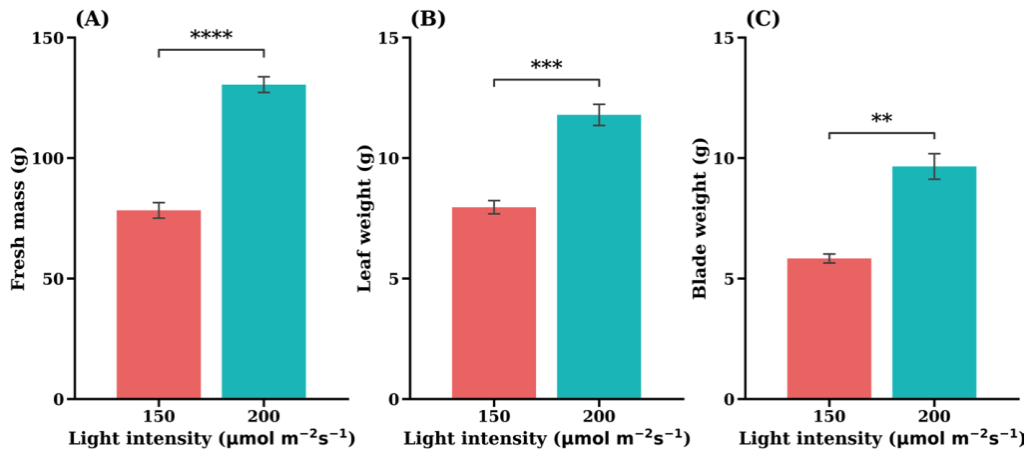
Effects of light intensity on fresh mass (**A**), leaf weight (**B**), and blade weight (**C**) in the iceberg lettuce cultivar ‘Celebration’. Values are expressed as the means ± SE (*n* = 5). Asterisks indicate significant differences between light intensity treatments according to Student’s *t*-test; ** *p* < 0.01; *** *p* < 0.001; **** *p* < 0.0001.

Light intensity had a statistically significant effect on the growth parameters of the iceberg lettuce cultivar. As shown in Figure 2, plants grown under 200 µmol·m^−2^·s^−1^ consistently exhibited greater biomass accumulation than those under 150 µmol·m^−2^·s^−1^. Specifically, the fresh mass (FM) reached approximately 130 g at 200 µmol·m^−2^·s^−1^, representing nearly a two-fold increase compared with ~78 g under the lower light treatment (Figure 2A). Similar trends were observed in leaf traits, with both leaf weight (Figure 2B) and blade weight (Figure 2C) being significantly higher under the higher light regime.

**Figure 3:**
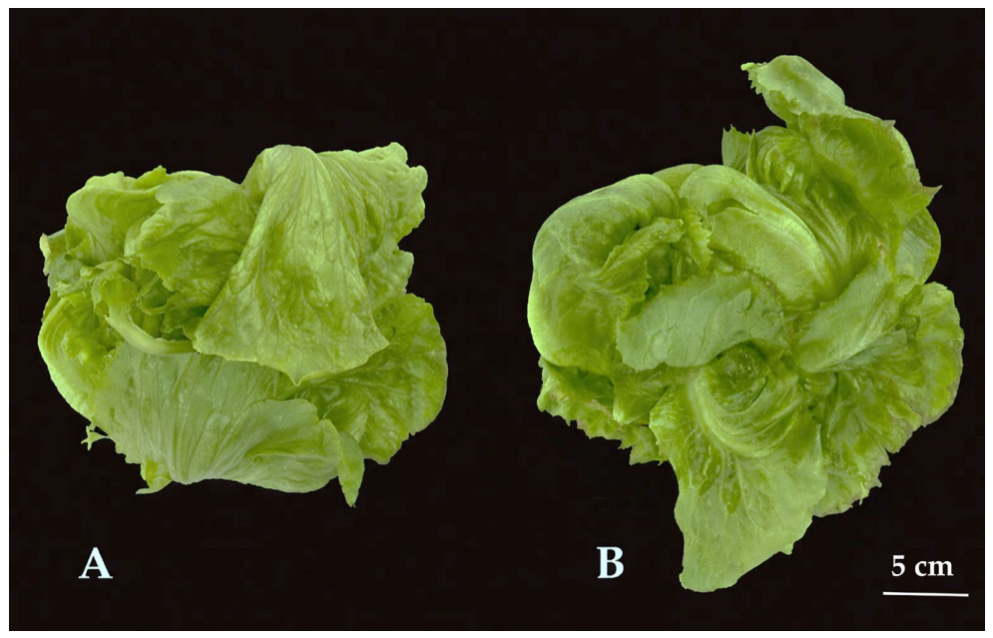
Morphology of the iceberg lettuce cultivar ‘Celebration’ grown under light intensity of 150 µmol·m^−2^·s^−1^ (**A**) and 200 µmol·m^−2^·s^−1^ (**B**). Scale bar = 5 cm.

These quantitative results are supported by the morphological differences observed in Figure 3. Under lower light intensity (150 µmol·m^−2^·s^−1^, Figure 3A), ‘Celebration’ formed tighter and more compact heads, albeit of smaller size. In contrast, plants grown under higher light intensity (200 µmol·m^−2^·s^−1^, Figure 3B) produced larger but looser heads, with outer leaves exhibiting reduced compaction.

**Figure 4:**
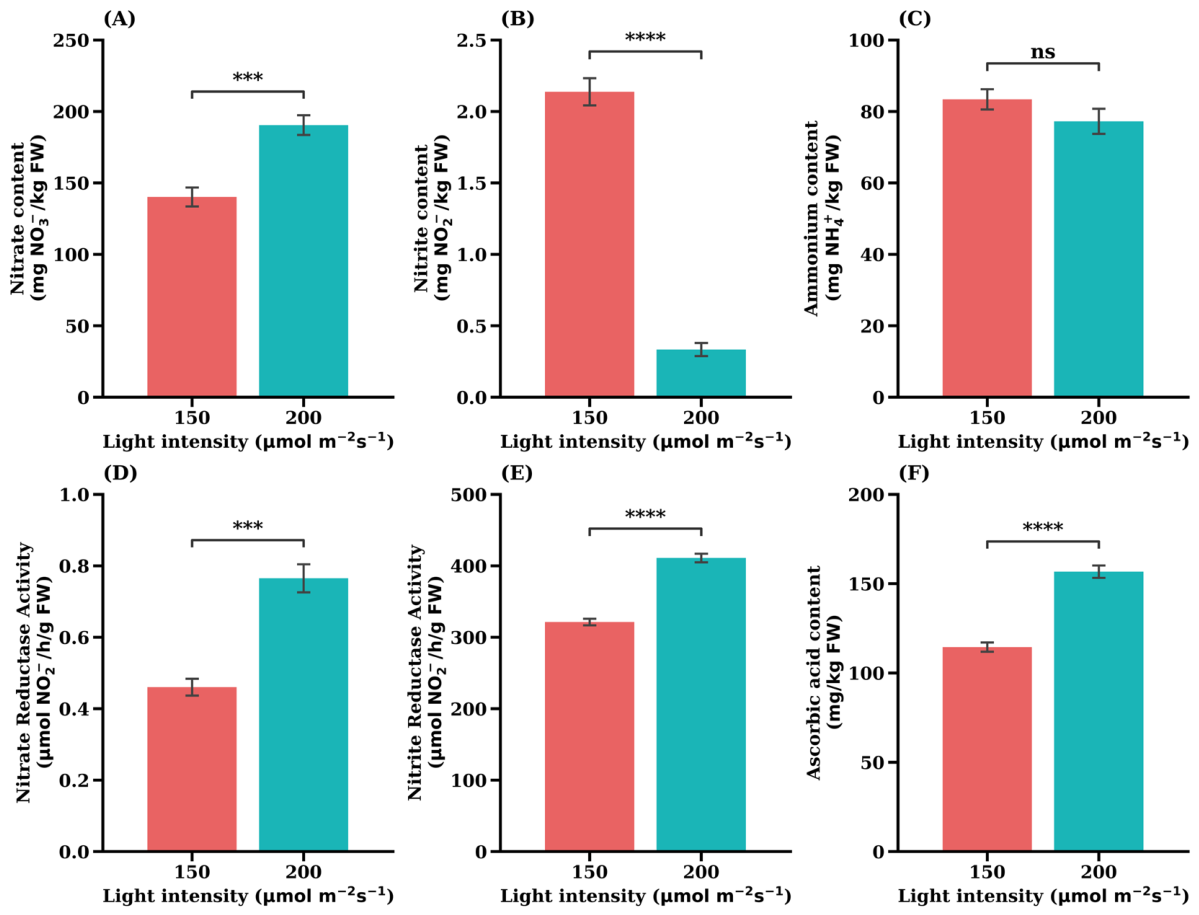
Effects of light intensity on nitrate content (**A**), nitrite content (**B**), ammonium content (**C**), nitrate reductase activity (**D**), nitrite reductase activity (**E**), and ascorbic acid (**F**) in the iceberg lettuce cultivar ‘Celebration’. Values are expressed as the means ± SE (*n* = 5). Asterisks indicate significant differences between light intensity treatments according to Student’s *t*-test: ns (not significant) *p* > 0.05; *** *p* < 0.001; **** *p* < 0.0001.

Exposure to higher light intensity (200 µmol·m^−2^·s^−1^) also resulted in significant changes in key biochemical and enzymatic parameters in the ‘Celebration’ cultivar, indicating a strong influence of the light environment (Figure 4). These findings suggest a regulatory shift in nitrogen assimilation and plant defense-related processes in response to light intensity.

Both NRA (Figure 4D) and NiRA (Figure 4E) significantly increased under the higher light intensity, indicating light-driven stimulation of the entire nitrogen reduction pathway. This enzymatic upregulation efficiently prevented the accumulation of toxic compounds, as demonstrated by the NO_2_^−^ content (Figure 4B), which decreased profoundly the under higher light condition and reached levels near zero. A reduction in NH_4_^+^ content was observed; however, this change was not statistically significant (Figure 4C, *p* > 0.05).

However, the enhanced enzymatic activity did not prevent the accumulation of the initial substrate, as NO_3_^−^ content (Figure 4A) significantly increased. This observation suggests that, under the higher light condition, nitrate uptake may be indirectly stimulated, as light intensity enhances photosynthetic carbon assimilation and increases plant nitrogen demand, thereby promoting nitrogen acquisition processes [13,20].

The increased synthesis of ascorbic acid at 200 µmol·m^−2^·s^−1^ (Figure 4F) (*p* < 0.0001) further highlights the plant’s enhanced metabolic state and defense mechanism, indicating a beneficial quality response.

**Figure 5:**
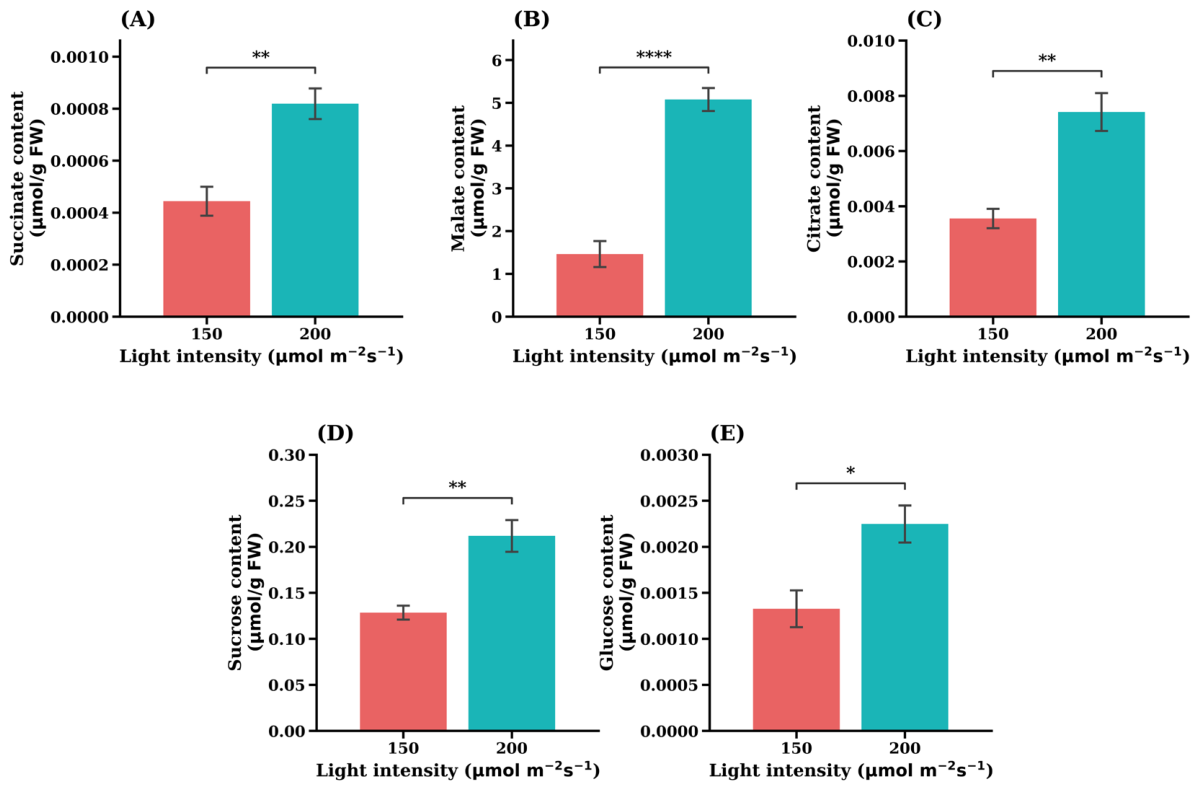
Increased carbohydrate metabolism under higher light intensity in the iceberg lettuce cultivar ‘Celebration’. (**A**) Succinate content, (**B**) Malate content, (**C**) Citrate content, (**D**) Sucrose content, (**E**) Glucose content. Values are expressed as the means ± SE (*n* = 5). Asterisks indicate significant differences between light intensity treatments according to Student’s *t*-test: * *p* < 0.05; ** *p* < 0.01; **** *p* < 0.0001.

**Figure 6:**
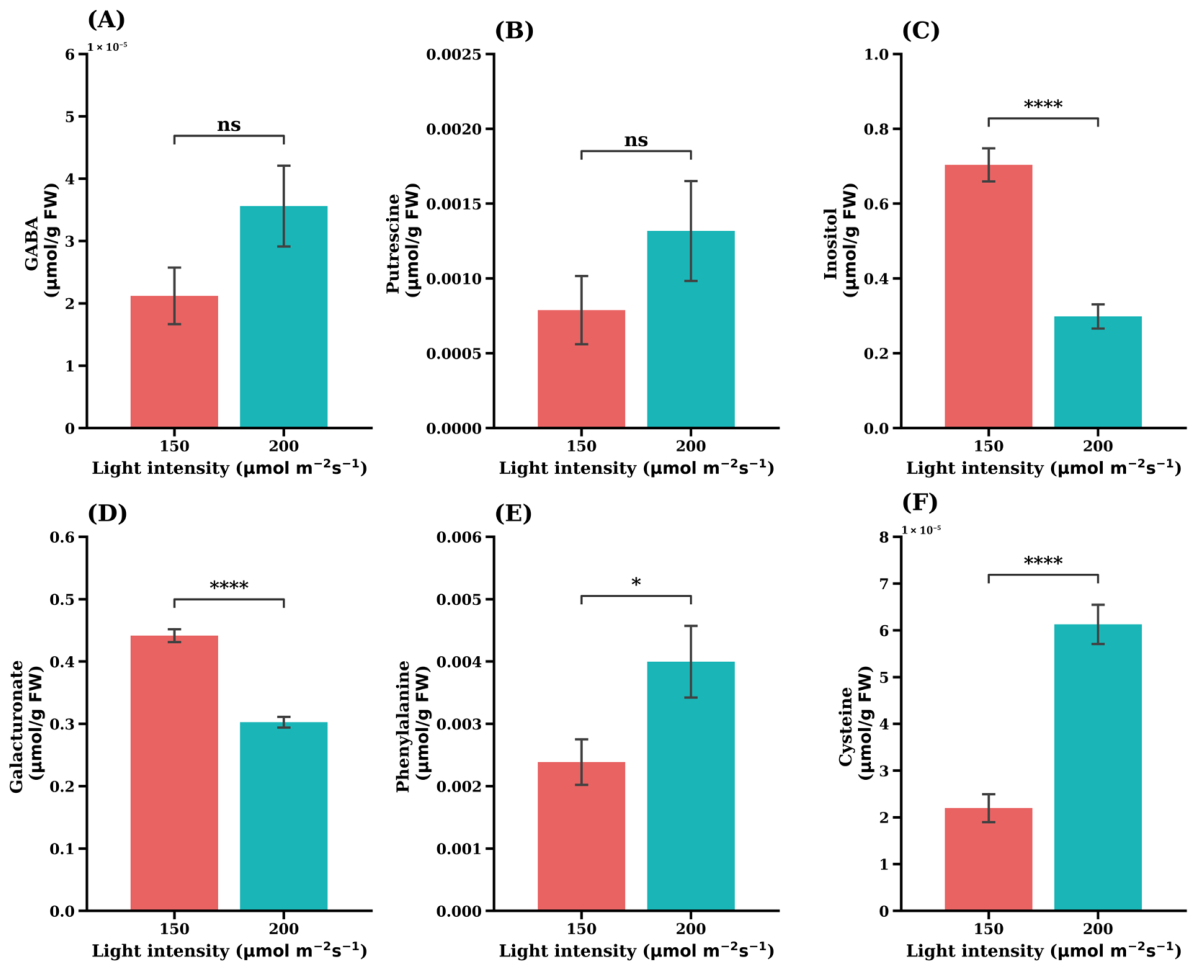
Effects of light intensity on GABA (**A**), putrescine (**B**), inositol (**C**), galacturonate (**D**), phenylalanine (**E**) and cysteine (**F**) in the iceberg lettuce cultivar ‘Celebration’. Values are expressed as the means ± SE (*n* = 5). Asterisks indicate significant differences between light intensity treatments according to Student’s *t*-test: ns (not significant) *p* > 0.05; * *p* < 0.05; **** *p* < 0.0001.

The metabolic responses of the iceberg lettuce cultivar ‘Celebration’ were examined under contrasting light intensities. Targeted metabolite analysis revealed significant alterations in several central carbon metabolites and related compounds. Specifically, TCA cycle intermediates (succinate, malate, and citrate) and soluble sugars (sucrose and glucose) showed marked increases under 200 µmol·m^−2^·s^−1^ (Figure 5). Similarly, amino acids such as phenylalanine and cysteine were also increased under this condition (Figure 6). In contrast, metabolites including inositol and galacturonate showed higher accumulation under 150 µmol·m^−2^·s^−1^ (Figure 6). These results indicate that light intensity strongly modulates carbon metabolism and associated metabolic pathways in ‘Celebration’ lettuce.

### 2.2. Effects of Light Intensity on Leaf Lettuce

**Figure 7:**
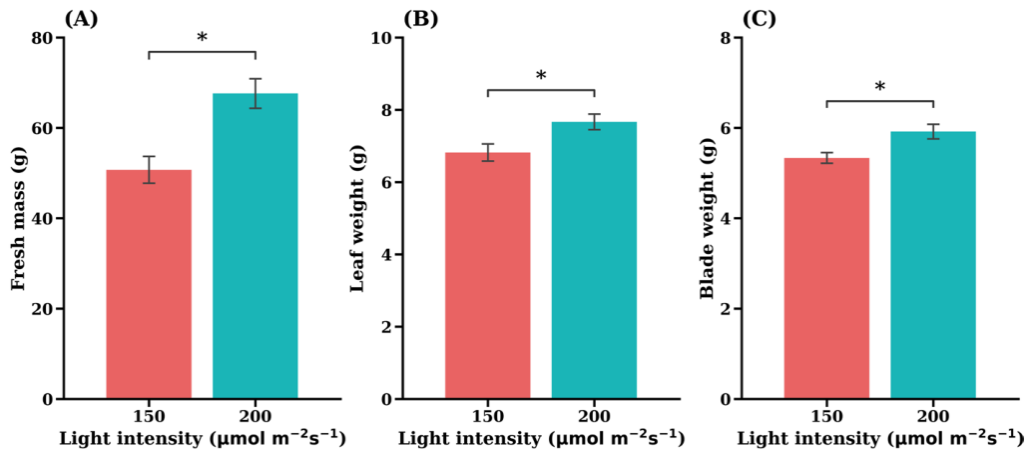
Effects of light intensity on fresh mass (**A**), leaf weight (**B**), and blade weight (**C**) in the leaf lettuce cultivar ‘Sunny’. Values are expressed as the means ± SE (*n* = 5). Asterisks indicate significant differences between light intensity treatments according to Student’s *t*-test:; * *p* < 0.05.

Light intensity had a significant effect on the growth performance of ‘Sunny’ leaf lettuce (Figure 7). Plants grown under 200 µmol·m^−2^·s^−1^ accumulated substantially greater fresh masses compared with those under 150 µmol·m^−2^·s^−1^ (Figure 7A) with an increase of approximately 25%. Similarly, both individual leaf weight (Figure 7B) and leaf blade weight (Figure 7C) were significantly higher under 200 µmol·m^−2^·s^−1^ (*p* < 0.05). Although the magnitude of increase in individual leaf components was smaller than that observed for total fresh mass, these results indicate that higher irradiance enhances biomass accumulation at both whole-plant and organ-specific levels.

**Figure 8:**
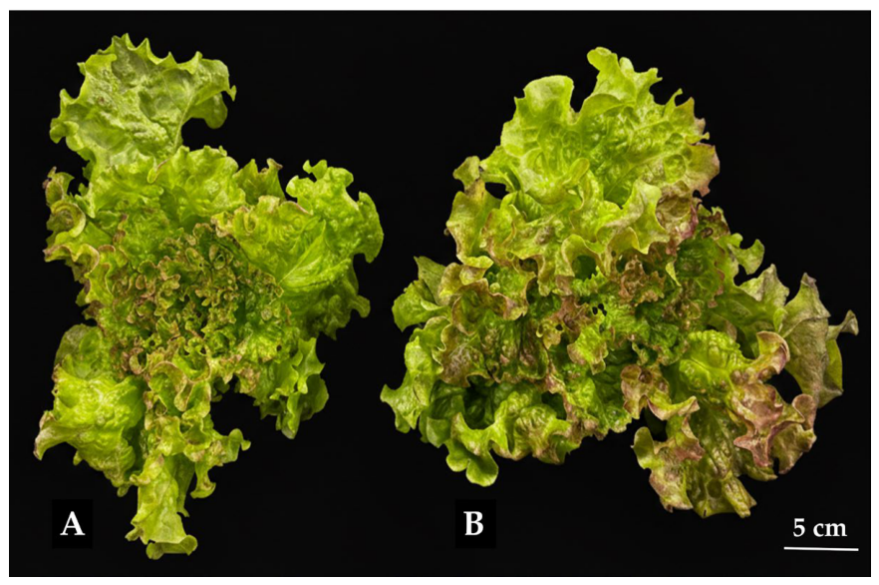
Morphology of the leaf lettuce cultivar ‘Sunny’ grown under light intensity of 150 µmol·m^−2^·s^−1^ (**A**) and 200 µmol·m^−2^·s^−1^ (**B**). Scale bar = 5 cm.

Morphological observations further supported these biomass trends (Figure 8). Under 150 µmol·m^−2^·s^−1^ (Figure 8A), leaves were moderately curled and less pigmented. In contrast, at higher light intensity (Figure 8B), ‘Sunny’ developed more expansive, turgid leaves and displayed enhanced red-purple pigmentation, particularly at the leaf margins.

**Figure 9:**
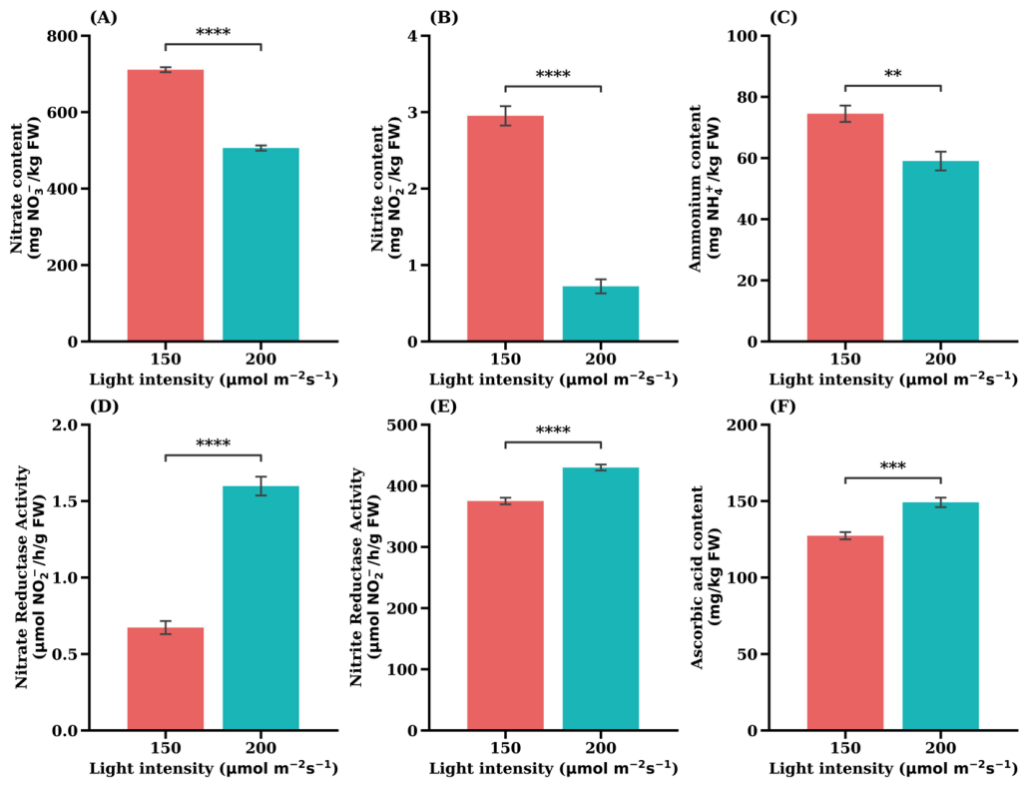
Opposite nitrate response in the leaf lettuce cultivar ‘Sunny’. (**A**) Nitrate content, (**B**) Nitrite content, (**C**) Ammonium content, (**D**) Nitrate reductase activity, (**E**) Nitrite reductase activity, (**F**) Ascorbic acid content. Values are expressed as the means ± SE (*n* = 5). Asterisks indicate significant differences between light intensity treatments according to Student’s *t*-test: ** *p* < 0.01; *** *p* < 0.001; **** *p* < 0.0001.

The comparative analysis of ‘Sunny’ lettuce revealed statistically significant effects across all six measured parameters when grown under 200 µmol·m^−2^·s^−1^ versus 150 µmol·m^−2^·s^−1^ (Figure 9). These results strongly suggest that the light environment modulates both nitrogen assimilation efficiency and key quality attributes in this cultivar.

The highest accumulation of major nitrogen compounds was observed under 150 µmol·m^−2^·s^−1^. Nitrate content (Figure 9A) was significantly higher under 150 µmol·m^−2^·s^−1^, averaging over 700 mg NO_3_^−^/kg FW compared with approximately 400 mg NO_3_^−^/kg FW under 200 µmol·m^−2^·s^−1^.

Similarly, nitrite (Figure 9B) (*p* < 0.0001) and ammonium (Figure 9C) (*p* < 0.01) contents were significantly elevated for the lower light condition. In contrast, both key assimilatory enzymes showed significantly higher activities under 200 µmol·m^−2^·s^−1^. NRA (Figure 9D) was strongly induced (*p* < 0.0001), while NiRA (Figure 9E) was also significantly increased (*p* < 0.01). The primary quality parameter, ascorbic acid content (Figure 9F), was significantly higher under higher light intensity (*p* < 0.001).

**Figure 10:**
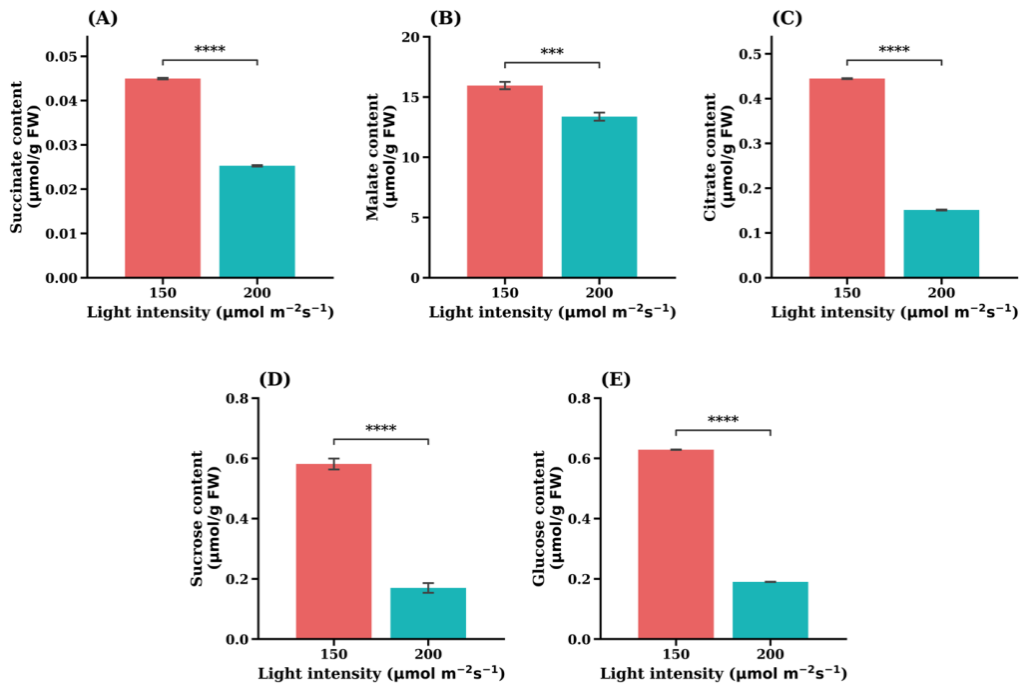
Opposite central carbon metabolite responses in the leaf lettuce cultivar ‘Sunny’. (**A**) Succinate content, (**B**) Malate content, (**C**) Citrate content, (**D**) Sucrose content, (**E**) Glucose content. Values are expressed as the means ± SE (*n* = 5). Asterisks indicate significant differences between light intensity treatments according to Student’s *t*-test: *** *p* < 0.001; **** *p* < 0.0001.

Light intensity significantly influenced the accumulation of central carbon metabolites in the leaf lettuce cultivar ‘Sunny’. As shown in Figure 10, the concentrations of the TCA cycle intermediate succinate (Figure 10A), malate (Figure 10B), and citrate (Figure 10C) were significantly higher under 150 µmol·m^−2^·s^−1^ than at 200 µmol·m^−2^·s^−1^. Among this, citrate exhibited the most pronounced response, showing a marked increase under reduced light intensity.

Similarly, soluble carbohydrate levels were strongly affected by light conditions. Both glucose (Figure 10D) and sucrose (Figure 10E) accumulated to significantly higher levels at 150 µmol·m^−2^·s^−1^ compared with 200 µmol·m^−2^·s^−1^. These results indicate that reduced irradiance promotes the accumulation of soluble sugars and several TCA-related organic acids in the ‘Sunny’ cultivar.

Overall, the metabolic profile suggests that carbon metabolites associated with energy metabolism and carbohydrate storage were preferentially accumulated under lower light conditions in ‘Sunny’ lettuce. This pattern contrasts with the response observed in nitrogen metabolism, highlighting a potential shift in carbon–nitrogen balance under different light regimes.

**Figure 11:**
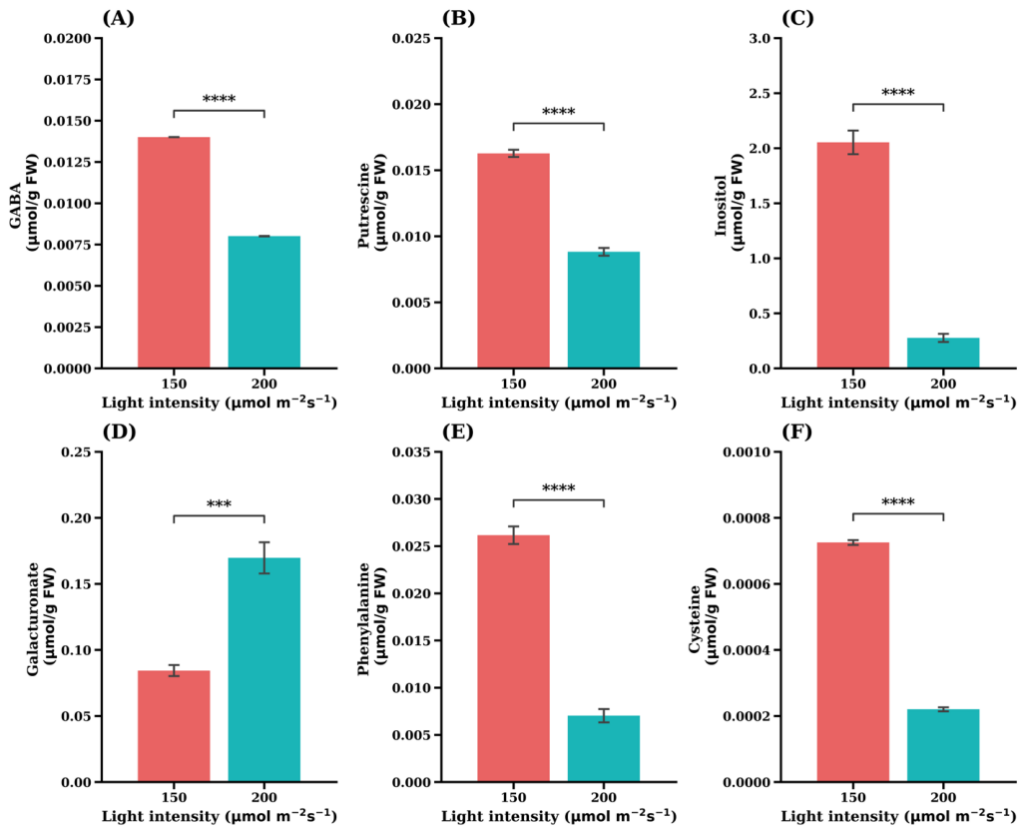
Accumulation in stress-related metabolites under low light intensity in the leaf lettuce cultivar ‘Sunny’. (**A**) GABA, (**B**) Putrescine, (**C**) Inositol, (**D**) Galacturonate, (**E**) Phenylalanine, (**F**) Cysteine. Values are expressed as the means ± SE (n = 5). Asterisks indicate significant differences between light intensity treatments according to Student’s *t*-test: *** *p* < 0.001; **** *p* < 0.0001.

In addition to central carbon metabolites, several amino acids and stress-related metabolites exhibited significant responses to light intensity. Under lower light conditions, the concentrations of GABA, putrescine, inositol, phenylalanine, and cysteine were significantly higher than those observed under higher light intensity (Figure 11). These metabolites are commonly associated with stress responses, osmotic regulation, and secondary metabolism.

In contrast, galacturonate (Figure 11D) showed the opposite pattern, with significantly higher concentrations under high light intensity. Galacturonate is associated with pectin metabolism and cell wall remodeling, suggesting that structural carbohydrate metabolism may respond differently to light availability compared with primary metabolic pathways.

Collectively, these results demonstrate that light intensity modulates multiple metabolic pathways in ‘Sunny’ lettuce, including central carbon metabolism, amino acid metabolism, and metabolites related to stress adaptation.

### 2.3. Correlation Network Linking Nitrogen Metabolism, Central Carbon Metabolites, and Growth Traits

**Figure 12:**
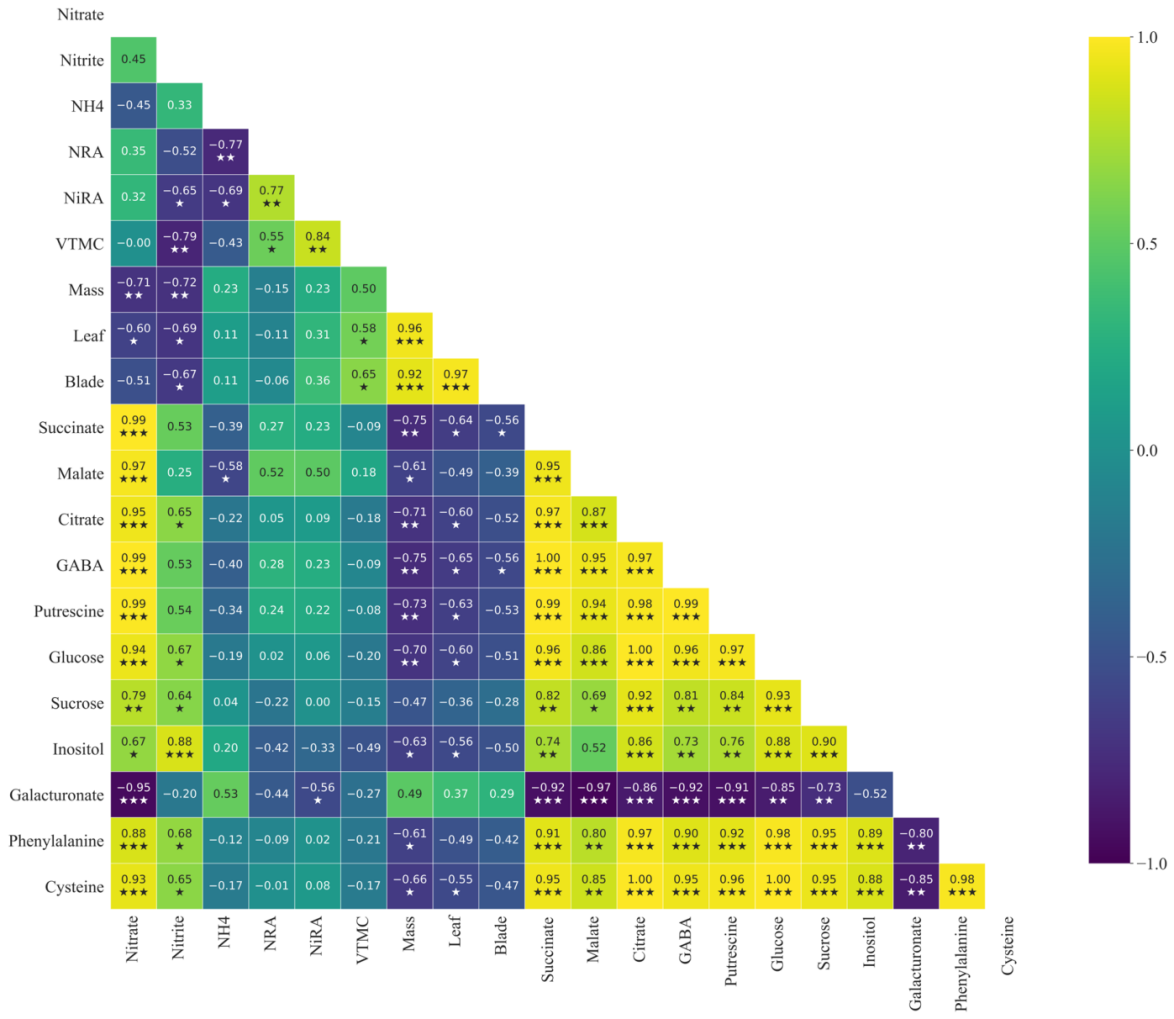
Pearson’s correlation heatmap illustrating the relationships among nitrogen metabolism parameters, primary metabolites, and growth traits in two lettuce cultivars, iceberg lettuce ‘Celebration’ and leaf lettuce ‘Sunny’, under different light intensity conditions. Correlation coefficients (r) were calculated using Pearson’s correlation analysis based on pooled data from all treatments and both cultivars. Positive correlations are represented by yellow–green colors, while negative correlations are shown in purple–blue according to the color scale. Color intensity reflects the strength of the correlation. Asterisks indicate statistical significance (* *p* < 0.05; ** *p* < 0.01; *** *p* < 0.001). Abbreviations: NRA: nitrate reductase activity; NiRA: nitrite reductase activity; NH_4_^+^: ammonium; VTMC: vitamin C content; Mass: fresh mass; Leaf: leaf weight; Blade: blade weight; GABA: γ-aminobutyric acid.

The enzymatic and metabolite analyses described above revealed clear responses of nitrogen metabolism and primary metabolites to light intensity. However, given that carbon metabolism, nitrogen assimilation, and plant growth are tightly interconnected processes, further analysis was conducted to examine the relationships among these variables. Pearson’s correlation analysis was performed using data from both cultivars and visualized as a correlation heatmap (Figure 12).

The heatmap revealed strong associations among inorganic nitrogen forms, nitrogen assimilation enzymes, primary metabolites, and growth-related traits. Nitrate concentration showed strong positive correlations with several organic acids and stress-related metabolites, including succinate (r = 0.99), malate (r = 0.97), citrate (r = 0.95), and GABA (r = 0.99), as well as with amino acids such as phenylalanine (r = 0.88) and cysteine (r = 0.93). It was also positively correlated with soluble sugars including glucose (r = 0.91) and sucrose (r = 0.79). In contrast, nitrate exhibited negative correlations with growth parameters, including fresh mass (r = −0.71), leaf weight (r = −0.60), and blade weight (r = −0.51).

Nitrite was positively associated with metabolites involved in osmotic regulation and carbon metabolism, such as inositol (r = 0.88), glucose (r = 0.67), and sucrose (r = 0.64), but negatively correlated with nitrate reductase (NRA) (r = −0.52) and nitrite reductase activities (NiRA) (r = −0.65). The activities of nitrate assimilation enzymes were strongly interrelated, with NRA positively correlated with NiRA (r = 0.77).

Growth-related traits were strongly intercorrelated, with fresh mass showing strong positive correlations with leaf weight (r = 0.96) and blade weight (r = 0.92). These traits were positively associated with vitamin C content (VTMC), suggesting that enhanced growth under favorable light conditions may be accompanied by enhanced antioxidant accumulation.

In contrast, galacturonate exhibited strong negative correlations with most central carbon metabolites and soluble sugars, indicating that cell wall-related metabolism may be regulated differently from primary carbon metabolism.

Overall, these relationships highlight a coordinated regulation between carbon and nitrogen metabolism, linking metabolic adjustments to growth performance under different light regimes.

## 3. Discussion

### 3.1. Physiological and Metabolic Adaptation of Iceberg Lettuce to Light Intensity

The positive correlation between light intensity and biomass accumulation observed in this study aligns with the fundamental principles of crop physiology, where light serves as the primary energy source driving photosynthesis and carbon assimilation. The substantial reduction in fresh mass under the lower light condition (Figure 2A) suggests that the photon flux density provided in the 150 µmol·m^−2^·s^−1^ experiment was insufficient to saturate the photosynthetic capacity of the iceberg variety, thereby limiting the production of photo assimilates required for organogenesis and expansion. These findings are consistent with recent studies on *Lactuca sativa*. For instance, Fan et al. (2013) reported that high irradiance increased the leaf dry weight of plants [33]. Similarly, Fu et al. (2017) reported that increasing light intensity from 60 to 220 µmol·m^−2^·s^−1^ significantly enhanced dry biomass in lettuce [34]. Our findings align closely with Voutsinos et al. (2021), who demonstrated that increasing PPFD from 188 to 310 µmol·m^−2^·s^−1^ significantly enhanced head size in vertical hydroponic lettuce [35]. A study on closed-type plant factories also corroborates that increasing irradiance in the range of 150–250 µmol·m^−2^·s^−1^ consistently increases yield but morphological quality parameters may not follow the same trend [36].

This pattern aligns with the morphological characteristics observed in iceberg cultivars when environmental conditions limit head-tightening processes. Iceberg lettuce typically requires cooler conditions (14–18 °C) to achieve firm head formation [36]. In our experiment, plants were maintained at 21 ± 1 °C with an extended photoperiod of 18 h light/6 h dark, conditions that are not optimal for head-tightening. These environmental parameters likely contributed to the looser head structure under high light, where increased irradiance accelerated growth but did not compensate for suboptimal temperature requirements for head formation.

When the plant receives sufficient light to sustain high photosynthetic rates, its demand for nitrogen, necessary for synthesizing essential proteins and amino acids, also rises significantly. This heightened demand triggers the roots to absorb NO_3_^−^ at a pace that exceeds the maximum conversion rate of the leaf tissue. Consequently, the excess NO_3_^−^ is temporarily stored, or “banked,” within the vacuoles of the leaf cells [34]. Therefore, the elevated NO_3_^−^ content serves as a key indicator of a highly active metabolic state geared towards rapid growth, reflecting the increased flux of nitrogen compounds through the plant system.

A positive correlation between the NR activity and free NO_3_^−^ content in plants was observed in this study (Figure 4A,D), which was in agreement with previous reports [35,36].

The safety analysis reveals that the NO_3_^−^ levels in the ‘Celebration’ lettuce under both lighting conditions remain well below the established regulatory limits. Even with the observed statistically significant increase in NO_3_^−^ content under 200 µmol·m^−2^·s^−1^ (rising to just below 200 mg/kg FW), the maximum measured concentration is considerably lower than the safety threshold of 2500 mg/kg FW [19]. This confirms that the nutritional solutions and light treatments employed resulted in a product that is safe for consumption.

Furthermore, the data indicate that higher irradiance stimulates the plant’s radical scavenging system, as evidenced by a significant increase in ascorbic acid (vitamin C) content under 200 µmol·m^−2^·s^−1^ (*p* < 0.0001). This enhanced production of a key antioxidant reflects a beneficial quality response to light-induced stress and is consistent with the finding of Zhang et al. (2018), who reported that higher PPFD promotes vitamin C accumulation [29]. Although internal nitrogen metabolism exhibited a degree of imbalance (characterized by elevated NiRA but reduced NH_4_^+^ levels), the plant effectively sequestered most nitrogen into non-toxic storage forms. These findings suggest that the higher light treatment, despite imposing a greater metabolic load, maintained a balance between yield potential and safety, ultimately producing lettuce with improved nutritional quality.

Taken together, these responses indicate that elevated light intensity induces a coordinated metabolic adjustment extending beyond antioxidant defense to central carbon metabolism. In the cultivar ‘Celebration’, higher light intensity significantly increased the accumulation of several TCA cycle intermediates (Figure 5), including succinate, malate, and citrate, suggesting enhanced carbon flux through respiratory pathways. This response is consistent with increased photosynthetic activity, where greater ATP and NADPH production supports CO_2_ assimilation and drives carbohydrate synthesis, leading to higher levels of soluble sugars such as sucrose and glucose [32,36]. These metabolites provide both energy and carbon skeletons required for downstream metabolic processes, ultimately resulting in increased levels of organic acids such as malate and citrate [37].

Interestingly, not all metabolites responded positively to increased light intensity. Inositol and galacturonate accumulated under lower light conditions (Figure 6), suggesting a shift toward cell wall-related and stress-associated metabolism when carbon availability is limited. Inositol plays key roles in membrane biosynthesis and stress signaling, while galacturonate is closely associated with pectin turnover and cell wall remodeling [38]. These findings indicate that light intensity reshapes metabolic allocation between growth-related processes and cellular maintenance pathways, consistent with previous metabolomic studies in lettuce [39,40,41,42].

Importantly, these changes in carbon metabolism were closely linked to nitrogen metabolism. Under higher light intensity, NRA and NiRA increased, accompanied by a reduction in NO_2_^−^ but a concurrent accumulation of NO_3_^−^ in leaf tissues. This pattern suggests that NO_3_^−^ uptake was stimulated to a greater extent than its downstream assimilation, as previously reported under high light conditions [13,23,34]. Despite this, NH_4_^+^ levels remained stable, indicating efficient metabolic regulation. The enhanced accumulation of TCA intermediates and soluble sugars likely provided sufficient carbon skeletons and energy to support rapid NH_4_^+^ assimilation via primary pathways (e.g., GS/GOGAT), as supported by studies on nitrogen assimilation in plants [37,43,44].

Furthermore, the increased levels of amino acids such as phenylalanine and cysteine indicate the active incorporation of inorganic nitrogen into organic forms, thereby preventing NH_4_^+^ accumulation. In contrast, the absence of significant changes in GABA and putrescine suggests that alternative NH_4_^+^ detoxification pathways were not strongly induced, consistent with their roles in stress and signaling responses rather than primary assimilation [45,46].

Collectively, these results demonstrate a tight coupling between carbon and nitrogen metabolism under elevated light intensity, whereby enhanced carbon flux supports efficient nitrogen assimilation, prevents NH_4_^+^ accumulation, and maintains metabolic balance despite increased NO_3_^−^ uptake. This coordinated regulation ultimately contributes to improved growth performance and nutritional quality in lettuce under optimized light conditions [27,29,47].

### 3.2. Physiological and Metabolic Adaptation of Leaf Lettuce to Light Intensity

Enhanced leaf expansion and coloration suggest improved photosynthetic performance and photoprotective mechanisms under elevated light intensity (Figure 8). Notably, the ‘Sunny’ cultivar did not exhibit head formation, which is consistent with its loose leaf morphological type.

Under higher light intensity (200 µmol·m^−2^·s^−1^), the ‘Sunny’ cultivar exhibited a visually more pronounced red-purple coloration compared with plants grown under lower light conditions. Although this observation is qualitative and was not supported by direct phenolic quantification, it is consistent with previous reports indicating that elevated photon flux density can stimulate anthocyanin accumulation in lettuce [48].

The enhanced growth observed in the present study is also consistent with earlier reports showing that increased light intensity promotes biomass accumulation in hydroponically grown lettuce. For example, Fu et al. (2017) reported that higher light intensity significantly increased lettuce biomass production [34]. Similarly, experiments using LED lighting demonstrated that shoot fresh weight increased with photosynthetic photon flux density (PPFD) up to approximately 300 µmol·m^−2^·s^−1^ [32]. Another study reported that lettuce grown under 200 µmol·m^−2^·s^−1^ exhibited leaf areas approximately 7–53% larger than those grown under lower intensities (100 and 150 µmol·m^−2^·s) accompanied by corresponding increases in fresh and dry shoot weights [49]. Comparable trends have also been observed across multiple lettuce types, including butterhead and Spanish-green cultivars [29,32,34,47,48]. Together, these findings support the conclusion that higher light intensity generally enhances photosynthetic activity, leaf expansion, and biomass accumulation in lettuce.

These studies complement our findings that high light intensity significantly enhances leaf mass and overall fresh weight in the ‘Sunny’ leaf lettuce cultivar (Figure 7), consistent with previous reports showing that increased irradiance promotes lettuce growth and biomass accumulation [27,32,36]. This consistency suggests that the underlying physiological responses—such as enhanced photosynthetic activity and increased leaf expansion under higher irradiance—are common mechanisms regulating lettuce growth across cultivars [32,33].

The results further indicate that the ‘Sunny’ cultivar efficiently utilizes higher light intensity to enhance nitrogen assimilation, thereby improving both growth performance and nutritional quality. Previous studies have reported that increased light intensity stimulates photosynthetic carbon assimilation and promotes nitrate reduction, resulting in lower NO_3_^−^ accumulation [27,29,34] and a higher vitamin C content [29,34]. Our findings are consistent with these observations.

The simultaneously elevated NRA (Figure 9D) and NiRA (Figure 9E) under high light intensity indicate that the nitrate reduction pathway operates at a greater capacity under these conditions. Nitrate reductase catalyzes the reduction of NO_3_^−^ to NO_2_^−^, while nitrite reductase subsequently converts NO_2_^−^ into NH_4_^+^, thereby completing the nitrate assimilation process [22,50]. The coordinated upregulation of these enzymes suggests the more efficient conversion of absorbed nitrate into reduced nitrogen forms that can be incorporated into amino acids and other nitrogen-containing metabolites.

Consistent with this interpretation, the concentrations of both NO_3_^−^ and NH_4_^+^ were lowest under high light intensity, indicating that absorbed nitrate was rapidly assimilated rather than accumulated. Under low light conditions, however, the activities of nitrate-assimilating enzymes were reduced, leading to slower nitrogen assimilation and the greater accumulation of inorganic nitrogen forms within plant tissues, which may be temporarily stored in cellular compartments when assimilation capacity is limited [20].

Importantly, even at the highest measured level under lower light conditions (below 800 mg kg^−1^ FW), the NO_3_^−^ concentration remained well below the maximum safety threshold of 4000 mg NO_3_^−^/kg FW established by the European Commission [19], indicating that lettuce produced under both lighting conditions is safe for human consumption.

Finally, the significant increase in ascorbic acid content observed under higher light intensity (Figure 9F) indicates an enhanced antioxidant response. Elevated irradiance can increase the production of reactive oxygen species in chloroplasts, and plants often respond by increasing the synthesis of antioxidant compounds such as ascorbic acid to maintain cellular redox homeostasis [51]. This response contributes not only to improved stress protection in plants but also to the enhanced nutritional quality of the harvested product.

These results indicate that elevated light intensity enhances the metabolic efficiency of ‘Sunny’ lettuce, resulting in lower accumulation of inorganic nitrogen forms and increased ascorbic acid content. Beyond these physiological responses, light intensity also exerts a strong regulatory effect on central carbon metabolism and associated metabolic networks.

Light intensity markedly influenced carbon metabolism in a cultivar-dependent manner. In the ‘Sunny’ cultivar, lower light intensity promoted the accumulation of several TCA cycle intermediates and soluble sugars, including citrate, succinate, glucose, and sucrose (Figure 10). These changes indicate a metabolic reprogramming of carbon metabolism in response to reduced irradiance.

Mechanistically, light availability regulates photosynthetic carbon assimilation and downstream metabolic fluxes. Under higher irradiance, increased ATP and NADPH production enhances CO_2_ fixation through the Calvin–Benson cycle, leading to greater synthesis of soluble carbohydrates that are subsequently metabolized through glycolysis and the TCA cycle to support energy production and biosynthesis. As these pathways are tightly interconnected, changes in carbon flux often led to substantial changes in organic acid and amino acid pools, reflecting the close integration between carbon and nitrogen metabolism in plants [37].

Under lower light conditions, reduced photosynthetic activity may limit the utilization for growth, resulting in the accumulation of soluble sugars and organic acids. Similar responses have been reported in leafy vegetables under nitrogen-related stress or metabolic imbalance, where organic acids and amino acids accumulate as part of adaptive metabolic adjustment [52,53]. The increased levels of citrate and other TCA intermediates observed here may therefore reflect altered respiratory flux and metabolic buffering under reduced irradiance.

In addition to primary metabolites, several stress-associated compounds, including GABA, putrescine, inositol, phenylalanine, and cysteine were significantly elevated under lower light intensity in the ‘Sunny’ cultivar (Figure 11). These metabolites are widely involved in stress response networks.

GABA functions as a signaling metabolite linking metabolic status to growth regulation [45], while polyamines such as putrescine contribute to stress tolerance by regulating redox balance and cellular balance [46]. Inositol also plays key roles in membrane synthesis and signaling pathways. Their accumulation suggests that lower irradiance induces metabolic adjustments associated with stress signaling and altered cellular homeostatic.

By contrast, galacturonate was higher under higher light conditions, indicating enhanced pectin turnover and cell wall metabolism [38], which is typically associated with active growth and leaf expansion under favorable energy conditions.

These metabolic responses observed in ‘Sunny’ lettuce were closely linked to nitrogen metabolism and plant performance. Under higher light intensity, ‘Sunny’ plants exhibited greater biomass accumulation along with increased NRA and NiRA, accompanied by lower concentrations of NO_3_^−^, NO_2_^−^, and NH_4_^+^ in leaf tissues. Similar interactions between light intensity and nitrogen metabolism have been reported in leafy vegetables [34,54,55,56]. Enhanced NO_3_^−^ assimilation likely contributed to improved nitrogen use efficiency and biomass production under higher light intensity [22,37].

Furthermore, improved metabolic performance under higher irradiance was associated with enhanced nutritional quality, as evidenced by increased ascorbic acid content. Ascorbic acid plays a central role in redox homeostasis and is positively associated with light-driven photosynthetic activity [51], with similar trends reported in controlled-environment studies [29,34].

Overall, these findings demonstrate that light intensity regulates lettuce metabolism through an integrated network linking carbon metabolism, nitrogen assimilation, and antioxidant production. Higher irradiance promotes efficient carbon fixation and nitrogen utilization, enhancing both yield and nutritional quality, whereas lower light induces the accumulation of stress-related metabolites and carbon reserves. This metabolic plasticity underscores the importance of optimizing light conditions in controlled-environment agriculture to maximize both productivity and crop quality.

### 3.3. Integrated Analysis of Metabolic Coordination and Plant Growth

The metabolite correlation network revealed a coordinated relationship between nitrogen metabolism, central carbon metabolism, and growth-related traits in lettuce. NO_3_^−^ exhibited strong associations with several soluble sugars and TCA cycle intermediates, including glucose, sucrose, malate, citrate, and succinate. These compounds represent key nodes in primary carbon metabolism and provide both energy and carbon skeletons required for nitrogen assimilation. The reduction of NO_3_^−^ to NH_4_^+^ and its subsequent incorporation into amino acids requires reducing power and organic carbon backbones such as 2-oxoglutarate, linking NO_3_^−^ metabolism directly to the activity of the TCA cycle and associated pathways [37]. The positive association between NO_3_^−^ and central carbon metabolites therefore likely reflects coordinated metabolic regulation in which enhanced carbon flux supports the energetic and biochemical requirements of nitrogen assimilation.

Similar metabolic interactions have been observed in metabolomic studies investigating plant responses to nitrogen stress, where adjustments in organic acid metabolism and amino acid synthesis contribute to maintaining carbon–nitrogen balance [37,52]. In these systems, increased flux through the TCA cycle supports nitrogen assimilation by supplying carbon skeletons and metabolic energy, thereby integrating carbon metabolism with nutrient utilization.

Beyond primary metabolism, several metabolites within the correlation network are associated with stress regulation and metabolic buffering. The accumulation of GABA, for instance, reflects the activation of the GABA shunt, a metabolic bypass connecting glutamate metabolism with the TCA cycle and contributing to the regulation of carbon–nitrogen balance under fluctuating environmental conditions [45]. Similarly, the presence of the polyamine precursor putrescine suggests the involvement of polyamine metabolism, which plays important roles in stress tolerance, membrane stabilization, and oxidative stress mitigation [46].

Another metabolite of particular interest is galacturonate, which is derived from the degradation of pectic polysaccharides in the plant cell wall. Pectin turnover is an important component of cell wall remodeling during plant growth and environmental adaptation, and galacturonate therefore reflects structural metabolic activity associated with cell expansion and tissue development [38]. The association between galacturonate and central carbon metabolites observed in the present study suggests potential links between primary metabolism and cell wall dynamics during lettuce growth.

Taken together, the metabolite correlations support a conceptual carbon–nitrogen metabolic network in which increased light availability enhances photosynthetic carbon fixation, elevating the supply of soluble sugars and organic acids that subsequently fuel nitrate assimilation and amino acid biosynthesis. Within this framework, metabolites associated with stress signaling, redox balance, and structural metabolism are integrated into the broader metabolic response. Similar integrated metabolic patterns have been reported in lettuce metabolomic studies, where coordinated regulation of primary and secondary metabolism was observed across different cultivars and environmental conditions [39,42].

While the correlation network provides a global view of the metabolic coordination between nitrogen assimilation, carbon metabolism, and plant growth, these relationships represent integrated responses across all treatments and cultivars. In reality, the strength and direction of these metabolic linkages can vary substantially depending on genotype-specific physiological strategies. Lettuce cultivars differ markedly in their capacity to balance carbon assimilation, nitrogen metabolism, and growth demand under changing light environments. Therefore, to better understand the biological mechanisms underlying the metabolic patterns observed in the correlation analysis, it is necessary to examine how individual cultivars modulate these pathways in response to light intensity.

In this context, the two lettuce cultivars investigated in this study exhibited distinct metabolic responses to irradiance. These differences provide an opportunity to further elucidate how the genotype-dependent regulation of carbon and nitrogen metabolism contributes to divergent physiological strategies in lettuce. The following section therefore examines the contrasting metabolic responses of the leaf lettuce cultivar ‘Sunny’ and the iceberg lettuce cultivar ‘Celebration’ under different light conditions.

### 3.4. The Contrasting Metabolic Responses to Light Intensity Between ‘Sunny’ Leaf Lettuce Cultivar and the ‘Celebration’Iceberg Lettuce Cultivar

Light intensity markedly influenced nitrogen metabolism in the two lettuce cultivars, but their responses differed substantially. In the leaf lettuce cultivar ‘Sunny’, NO_3_^−^ content decreased significantly under higher light intensity, whereas in the iceberg lettuce cultivar ‘Celebration’ NO_3_^−^ concentrations increased. This contrasting behavior can be explained by differences in canopy architecture and the balance between nitrate uptake and assimilation. ‘Sunny’ forms loosely arranged leaves that allow deeper and more uniform light penetration throughout the canopy. Under high irradiance, the enhanced NRA effectively converts absorbed NO_3_^−^ to NO_2_^−^, thereby limiting NO_3_^−^ accumulation in plant tissues.

In contrast, ‘Celebration’ develops a compact head structure with densely layered leaves. Even under higher light conditions, inner leaf tissues may remain partially shaded, reducing the effective upregulation of NO_3_^−^ reduction capacity in deeper tissues. At the same time, higher irradiance likely stimulates photosynthesis and increases nitrogen demand for amino acid and protein synthesis, thereby enhancing NO_3_^−^ uptake. When NO_3_^−^ uptake exceeds the capacity for reduction, NO_3_^−^ accumulates in leaf tissues despite increased light availability. Similar mechanisms have been reported in lettuce, where internal shading or dense canopy structures limit NO_3_^−^ assimilation and promote NO_3_^−^ accumulation [57,58]. Genotype-dependent variation in NO_3_^−^ accumulation among lettuce types has also been documented in previous studies, demonstrating that cultivar characteristics strongly influence nitrogen metabolism even under uniform cultivation conditions [59,60].

Despite these differences, NO_3_^−^ concentrations observed in both cultivars remained well below regulatory limits for leafy vegetables. The maximum NO_3_^−^ content measured in ‘Sunny’ (approximately 700 mg NO_3_^−^ kg^−1^ FW) and ‘Celebration’ (approximately 140 mg NO_3_^−^ kg^−1^ FW) was substantially lower than the maximum levels permitted for lettuce in commercial production systems [19].

NH_4_^+^ concentrations also responded differently to light intensity between the two cultivars. In ‘Sunny’, NH_4_^+^ levels were significantly higher under low light conditions, whereas ‘Celebration’ showed no significant difference between light treatments. NH_4_^+^ assimilation primarily occurs through the glutamine synthetase/glutamate synthase (GS/GOGAT) pathway, which requires both reducing power and carbon skeletons derived from photosynthetic metabolism [43,44]. Under reduced light intensity, limited carbon availability may constrain the incorporation of NH_4_^+^ into amino acids, resulting in its accumulation within plant tissues [37,41]. Similar interactions between carbon metabolism and NH_4_^+^ assimilation have been widely reported in plant nitrogen metabolism studies [37,44]. The relatively stable ammonium levels observed in ‘Celebration’ across light treatments may suggest tighter metabolic regulation or differences in nitrogen partitioning associated with cultivar-specific physiological characteristics.

Light intensity also significantly affected ascorbic acid accumulation in both cultivars, with higher concentrations observed under high light conditions. Ascorbic acid plays a crucial role in plant antioxidant defense and redox regulation, particularly under elevated irradiance when reactive oxygen species production increases in chloroplasts. Increased ascorbate accumulation under high light therefore reflects the activation of photoprotective mechanisms that maintain cellular redox balance and protect metabolic enzymes involved in photosynthesis and nitrogen assimilation. Similar responses have been reported in leafy vegetables grown under controlled environments [29,58].

These physiological and biochemical differences between the two cultivars are further reflected in their broader metabolic profiles. A notable outcome of the present study is the contrasting metabolic responses of the two lettuce cultivars to light intensity, highlighting the clear genotype-dependent regulation of metabolic pathways. While the iceberg cultivar ‘Celebration’ showed enhanced accumulation of central carbon metabolites under high light conditions, the leaf lettuce cultivar ‘Sunny’ exhibited the opposite trend, with higher levels of several TCA cycle intermediates and soluble sugars under low light intensity.

These divergent patterns may reflect differences in how the two cultivars balance carbon assimilation, respiratory metabolism, and growth demand. In ‘Celebration’, higher irradiance likely enhanced photosynthetic carbon fixation and increased the supply of photoassimilates entering glycolysis and the TCA cycle, resulting in the greater accumulation of organic acids and soluble sugars. In contrast, ‘Sunny’ appears to utilize photoassimilates more rapidly for biomass production and nitrogen assimilation under high light conditions, leading to lower steady-state concentrations of carbon intermediates despite enhanced photosynthetic activity.

Such responses can be interpreted within a source–sink regulatory framework. Increased irradiance strengthens photosynthetic source activity by generating larger pools of carbon assimilates. In ‘Sunny’, the open canopy structure and efficient nitrate reduction may facilitate the rapid integration of carbon and nitrogen metabolism, directing carbon skeletons toward amino acid synthesis and growth. Conversely, the dense leaf architecture of ‘Celebration’ may create spatial heterogeneity in light availability and metabolic activity within the canopy, allowing carbon and nitrogen intermediates to accumulate in tissues where metabolic demand is lower.

These findings are consistent with metabolomic studies demonstrating strong genotype-dependent variation in metabolic pathway activity and metabolite accumulation patterns in lettuce [39,42]. Genetic background influences not only biochemical regulation but also plant structural traits that determine internal light distribution and metabolic activation.

Overall, the results indicate that light acts as a central regulator integrating carbon metabolism, nitrogen assimilation, and antioxidant responses in lettuce. However, the efficiency with which these processes are coordinated depends strongly on cultivar-specific physiological and structural characteristics. Such genotype-dependent metabolic strategies ultimately determine nitrogen utilization efficiency, biomass production, and nutritional quality in controlled-environment lettuce cultivation.

## 4. Materials and Methods

### 4.1. Plant Materials and Growth Conditions

Seeds of the iceberg lettuce cultivar ‘Celebration’ (var. *capitata* L. *nidus jaggeri* Helm) sourced from Sakata Seed Co., Yokohama, Japan, and the leaf lettuce cultivar ‘Sunny’ (var. *acephala* Alef., syn. var. *secalina* Alef., syn. var. *crispa* L.) obtained from Tohoku Seed Co., Tokyo, Japan, were propagated in a plastic seedling tray containing watered hydroponic rockwool cubes (36 mm × 36 mm × 40 mm). Following germination, the seedlings were cultivated in a closed plant system that featured a photoperiod of 18 h of light and 6 h of darkness at a temperature of 21 ± 1 °C, with a relative humidity of 70 ± 10% at as outlined by Jeong et al. (2013) [61]. Light-emitting diodes (LEDs) (T8 LED tube, Nichia, Anan, Japan) were installed horizontally at a height of 40 cm above the work surface to serve as the light source. The PPFDs were assessed at the top of the plants using a Light Analyzer LA-105 (Nippon Medical & Chemical Instruments Co., Ltd., Osaka, Japan). The light source treatments were set to two different light intensities of 150 and 200 µmol·m^−2^·s^−1^. These varying levels of light intensity were achieved by positioning the cultivation beds on shelves of various heights within the same closed plant system. The spectral distribution of the lamps was measured across light wavebands from 380 nm to 780 nm, and the average values collected from three distinct locations within the plant growing area under light treatment are presented in Table 1.

When the seedlings had 5 true leaves, they were transplanted to the hydroponic cultivation bed. The conditions remained unchanged until harvest. The cultivation bed comprised a PP cultivation bench with a dimension of 570 mm (L) × 385 mm (W) × 100 mm (H) along with an XPS board that had a thickness of 27 mm, serving as a bench cover equipped with twelve planting holes, each having a diameter of 50 mm.

The lettuce plants received a nutrient solution consisting of liquid fertilizers Hyponica A and Hyponica B, mixed in a ratio of 20 mL of Hyponica A to 20 mL of Hyponica B in 10 L of water, which served as the standard solution (Kyowa Co., Ltd., Japan). The NPK formulation of Hyponica liquid fertilizer is designed with a nitrogen (N) ratio of 4, phosphorus (P) ratio of 3.8, and potassium (K) ratio of 9.4, along with a balanced mixture of essential elements for plant growth, including calcium, magnesium, sulfur, iron, boron, copper, zinc, and molybdenum. The pH of the nutrient solution was checked daily with a calibrated pH meter and maintained between 5.5 and 6.0 by adjusting with diluted phosphoric acid or potassium hydroxide as needed, while the electrical conductivity (EC) was maintained at 1.2 to 1.4 mS/cm. Only pure water was utilized for the first four days after seeding. After germination, a quarter strength of the standard solution was applied during the cotyledon phase, and one-third strength was used during the stage with one to two true leaves. Once the lettuce exhibited two to three true leaves, a half-strength nutrient solution was administered. When the seedlings achieved five true leaves, they were transplanted to the hydroponic cultivation bed utilizing a full-strength nutrient solution [29]. The nutrient solution was refreshed every seven days.

The experiments were conducted on three separate occasions: from 9 September to 28 October 2024, from 1 December to 18 January 2025, and from 1 April to 18 May 2025. Since the results were consistently similar, the data presented in this paper are derived from the third experiment.

After thirty days of transplanting, the lettuce plants were harvested and weighed to define the fresh mass using lab scales and expressed in gram. The third and/or fourth leaves, being the largest fully expanded leaves, were collected as samples for the assessment of leaf weight, blade weight and biochemical components such as nitrate, nitrite, ammonium, and ascorbic acid, as well as for the analysis of enzyme activity including nitrate and nitrite reductase, conducted on the same day.

### 4.2. Nitrate and Nitrite Content

The nitrate and nitrite contents in vegetables were determined using the reflectometric method after a reducing agent and Griess reaction [62] using a reflectometer (RQ-flex Plus 10, Merck Inc., Darmstadt, Germany) according to a prior study [12]. The nitrate and nitrite contents were expressed as mg NO_3_^−^/kg fresh weight (FW) and mg NO_2_^−^/kg FW respectively.

### 4.3. Ammonium Content

Ammonium content in the crude extract was determined using the modified Berthelot reaction as described by Weatherburn in 1967 [63]. A sample of 0.5 g of lettuce blade was ground with a mortar and pestle in the presence of 3 mL of 0.3 mM sulfuric acid, maintaining a pH of 3.5. The resulting homogenate underwent centrifugation at 12,000 rpm for a duration of 10 min at a temperature of 25 °C. Subsequently, 200 µL of the clear supernatant was diluted with 3.8 mL of 0.3 mM sulfuric acid to achieve a total volume of 4 mL. For the colorimetric reaction, 0.5 mL of solution A, which comprises 5 g of phenol and 25 milligrams of nitroprusside dissolved in 100 mL of water, was added, followed by the addition of 0.5 mL of solution B. Solution B was created by mixing 40 mL of 5% sodium hypochlorite with 2.5 g of NaOH and diluting to a final volume of 100 mL with distilled water. The mixture was allowed to incubate with gentle shaking in a water bath set at 37 °C for 20 min. The absorbance was then recorded at 625 nm in comparison to a control that did not contain the extract. The levels of ammonium were determined using an extinction coefficient of 3.646 µmol^−1^·cm^−1^ and were reported as mg NH_4_^+^ per kg of fresh weight.

### 4.4. Vitamin C Content

The vitamin C (ascorbic acid) contents in the vegetables were determined using a reflectometric method, using a reflectometer (RQ-flex Plus 10, Merck Inc., Darmstadt, Germany) according to a prior study [12]. Ascorbic acid content was expressed as mg per kg of fresh weight.

### 4.5. Nitrate Reductase Activity

The activity of nitrate reductase (NR) was measured following the protocol proposed by Neyra and Hageman (1974) [50] with modifications as indicated by Segura (1990) [64].

The tissue of 0.5 g from the third leaves was collected, finely chopped, and then placed in 5 mL of incubation solution with a pH of 7.5. This was done in a dark environment at room temperature, specifically at 28 ± 2 °C, for a duration of 60 min. The incubation solution consisted of 1 mL of 0.1 M KNO_3_, 3.75 mL of a mixture of 0.1 M K_2_HPO_4_ and KH_2_PO_4_, along with 0.25 mL of 1% (*v*/*v*) n-propanol. After the incubation, new solutions were created using 2 mL of the incubation solution combined with 1 mL of nitrite reactive (Sulfanilamide at 1% (*w*/*v*) in HCl 3 M and 0.02% N-(1-naftil) ethylene di-amine di-hydrochloride) and a control consisting of 2 mL of the incubation solution with 1 mL of distilled water. This mixture was then incubated in darkness for 15 min to allow for color development. The absorbance was measured using a spectrophotometer at a wavelength of 540 nm to generate a nitrite standard curve with a 10 ppm N solution (as NaNO_2_). The results were expressed as one unit of NR activity in µmol of NO_2_^−^ per hour per gram of fresh weight.

### 4.6. Nitrite Reductase Activity

Nitrite reductase (NiR) activity was measured following the methodology established by J. L. Wray & R. J. Fido in 1990 [65], which involved using dithionite-reduced methyl viologen as an artificial electron donor.

About 0.5 g of the fresh sample was crushed in a pre-chilled mortar with a pestle containing 5 mL of distilled water. Subsequently, the extract was subjected to centrifugation at a speed of 12,000 rpm for 10 min at a temperature of 25 °C utilizing the TOMY MX-307 (Tomy Kogyo Co., Ltd., Tokyo, Japan) high-speed refrigerated microcentrifuge from Japan. For the assay, 50 µL of the extract was combined with 125 µL of 50 mM potassium phosphate buffer (pH 7.5), 125 µL of potassium nitrite (2.5 mM KNO_2_) and 200 µL of freshly prepared 20 mM sodium dithionite Na_2_S_2_O_4_ in 290 mM sodium bicarbonate. The reaction was initiated with the addition of sodium dithionite, followed by the incorporation of 125 µL of methyl viologen (3 mM methyl viologen) to develop a blue color. Control samples, referred to as blanks, included all components of the assay with the exception of methyl viologen. After allowing incubation for the specified time period of 15 min at 25 °C in open tubes, the reaction was halted by thoroughly mixing the contents of the tube until both dithionite and the reduced methyl viologen were oxidized, which was evidenced by the disappearance of the blue color of the reduced dye. A 0.1 mL sample of the reaction mixture was diluted with 2.9 mL of distilled water, along with 1 mL of each of the 1% (*w*/*v*) sulphanilamide in 3 M HCl and 0.02% (*w*/*v*) N-(1-naphthyl) ethylene-diamine dihydrochloride. This mixture was then incubated for an additional 15 min. The absorbance was recorded at a wavelength of 540 nm using an appropriate blank in a spectrophotometer, which facilitated the calculation of NiR activity. One unit of NiR activity was defined as the production of 1 μmol of NO_2_^−^ per hour per gram of fresh weight.

### 4.7. Metabolite Analysis

Metabolic profiling of lettuce was conducted using gas chromatography–mass spectrometry (GC–MS). For metabolite extraction, 100 mg of freeze-dried and homogenized lettuce powder was transferred into a 2 mL microcentrifuge tube containing one zirconia bead and 250 µL of methanol. The samples were homogenized using a Mixer Mill MM400 (Retsch GmbH, Haan, Germany) for 2 min at 27 Hz.

Subsequently, 250 µL of chloroform was added, and the mixture was incubated in a Thermomixer F2.0 (Eppendorf AG, Hamburg, Germany) for 3 min at 37 °C and 1200 rpm. After incubation, 50 µL of internal standard solution (ribitol, 2 mg mL^−1^ in ultrapure water) and 175 µL of ultrapure water were added to each sample. The mixture was vortexed thoroughly using a Vortex Genie 2 (Scientific Industries Inc., Bohemia, NY, USA) to ensure complete mixing.

The samples were centrifuged at 1200 rpm for 10 min at 25 °C using a MX-307 centrifuge (TOMY Seiko Co., Ltd., Tokyo, Japan). Following centrifugation, 80 µL of the supernatant was transferred to a new 1.5 mL microcentrifuge tube and subjected to centrifugal evaporation for 2 h using a CVE-3110 centrifugal evaporator (EYELA, Tokyo Rikakikai Co., Ltd., Tokyo, Japan). The dried extracts were subsequently freeze-dried overnight at −45 °C using a freeze dryer (FDM-100, EYELA, Japan).

For chemical derivatization, 40 µL of methoxyamine hydrochloride (MACl) solution (20 mg MACl dissolved in 1 mL pyridine) was added to each sample. The samples were incubated in the Thermomixer F2.0 for 90 min at 37 °C to allow methoximation. Subsequently, 50 µL of N-methyl-N-trimethylsilyl trifluoroacetamide (MSTFA) was added for silylation. The samples were briefly centrifuged using a C1008-B centrifuge (Nichiryo Co., Ltd., Tokyo, Japan) and incubated again in the thermomixer at 37 °C for 30 min.

Following derivatization, 50 µL of the final solution was transferred into GC–MS vials for metabolite analysis. GC–MS analysis was performed using a GC-2010 gas chromatograph coupled with a GCMS-QP2010 Plus mass spectrometer (Shimadzu Corporation, Kyoto, Japan). Metabolite separation was achieved using a DB-5MS capillary column (30 m × 0.25 mm internal diameter × 1.00 µm film thickness; Agilent Technologies, Santa Clara, CA, USA).

The GC operating conditions were as follows: the inlet temperature was set at 280 °C, and samples were injected in split mode (10:1). The oven temperature was initially held at 100 °C for 1 min, increased to 320 °C at a rate of 4 °C min^−1^, and maintained at 320 °C for 10 min. Helium was used as the carrier gas at a constant flow rate of 1.1 mL min^−1^.

The mass spectrometer was operated in electron ionization (EI) mode with scan acquisition. The transfer line temperature was maintained at 280 °C, and the ion source temperature was set at 200 °C. Mass spectra were recorded at a scan rate of 1 scan s^−1^ over a mass-to-charge ratio (*m*/*z*) range of 45–600.

Chromatographic peak detection, deconvolution, and integration were performed using GCMSsolutionv2.49 software (Shimadzu Corporation, Kyoto, Japan). Metabolite identification was achieved by comparing the obtained mass spectra with reference spectra from the NIST Mass Spectral Library (National Institute of Standards and Technology, Gaithersburg, MD, USA). Compound identification was based on spectral similarity and retention time consistency.

For semi-quantitative analysis, metabolite peak areas were normalized to the internal standard (ribitol) to correct for variations in extraction efficiency and instrumental response. The normalized peak areas were used for relative comparison of metabolite abundance among treatments.

### 4.8. Statistical Analysis

All experiments were conducted in triplicate, and results are presented as mean ± standard error (SE). Data were tested for normality and homogeneity of variance prior to analysis. Normality was assessed using the Shapiro–Wilk test and confirmed by visual inspection of histograms and Q–Q plot (*p* > 0.05). Differences between light intensity treatments within each lettuce cultivar, as well as between 150 µmol·m^−2^·s^−1^ and 200 µmol·m^−2^·s^−1^ across cultivars, were evaluated using Student’s *t*-test. Correlation coefficients (r) were calculated using Pearson’s correlation analysis based on pooled data from all treatments and both cultivars. Statistical analyses were performed using R (v4.4.1, R-Studio v2025.05.1+513). Data visualization was conducted using Python3.12.13 (Google Colab) and correlation heatmaps were generated using Jupyter Notebook v7.5.4.

## 5. Conclusions

Light availability acts as a key environmental regulator linking carbon metabolism, nitrogen assimilation, and antioxidant responses in lettuce cultivated under controlled environments. Increasing light intensity enhanced NRA and NiRA in both cultivars, confirming that higher irradiance stimulates the biochemical capacity for NO_3_^−^ reduction. However, the final outcome of NO_3_^−^ accumulation was strongly cultivar-dependent.

The leaf lettuce cultivar ‘Sunny’ effectively translated enhanced NO_3_^−^ reduction into lower NO_3_^−^ accumulation under higher light conditions. In contrast, the iceberg lettuce cultivar ‘Celebration’ exhibited increased NO_3_^−^ levels under the same treatment. This contrasting response likely reflects structural differences between cultivars. The compact head architecture of iceberg lettuce may limit internal light penetration within densely layered leaves, thereby restricting NO_3_^−^ reduction in inner tissues despite increased external irradiance.

Beyond nitrogen metabolism, elevated irradiance also promoted ascorbic acid accumulation, indicating the activation of photoprotective antioxidant systems. Metabolomic analysis further revealed cultivar-specific differences in central carbon metabolism and stress-related metabolites, suggesting differential coordination between photosynthetic carbon assimilation, respiratory metabolism, and nitrogen utilization.

Taken together, these findings demonstrate that environmental light conditions interact with genotype-specific structural and physiological traits to determine metabolic balance, nitrogen use efficiency, and crop quality in lettuce grown under controlled environments.

### 5.1. Practical Implications

In PFALs, lettuce is typically cultivated under PPFDs of approximately 150–250 µmol·m^−2^·s^−1^, a range designed to balance biomass production with energy use efficiency in indoor cultivation systems [29]. The present results indicate that moderate increases in light intensity within this practical range can enhance NO_3_^−^ assimilation capacity and antioxidant accumulation. However, the magnitude and direction of nitrate responses remain cultivar-dependent. These findings suggest that lighting strategies in PFAL systems should be optimized in combination with cultivar selection in order to simultaneously improve productivity, NO_3_^−^ safety, and nutritional quality in hydroponic lettuce production.

### 5.2. Future Perspectives

Although this study provides insight into metabolic responses to light intensity, the analysis was based primarily on steady-state metabolite profiles. Future research integrating metabolic flux analysis, enzyme activity measurements, and transcriptomic approaches would help clarify how carbon and nitrogen fluxes are dynamically coordinated under different light regimes. Expanding comparative studies across a broader range of lettuce genotypes and environmental variables, including temperature interactions and light spectral composition, will further advance our understanding of metabolic plasticity in controlled-environment agriculture.

## Figures and Tables

**Table 1:** Spectral characteristics of the LEDs.

Light Source	Photosynthetic Photon Flux Density (µmol·m^−2^·s^−1^ PPFD)				
	380–400 nm	400–500 nm	500–600 nm	600–700 nm	700–780 nm
LED tube *	0.4	65.8	47.5	149.9	4.4

* 0.5 W per LED chip.

## Data Availability

The raw data supporting the conclusions of this article will be made available by the authors on request.

## Abbreviations

The following abbreviations are used in this manuscript:

NRA	Nitrate Reductase Activity
NiRA	Nitrite Reductase Activity
NO_3_^−^	Nitrate
NO_2_^−^	Nitrite
NH_4_^+^	Ammonium
PPFD	Photosynthetic Photon Flux Density
PFAL	Plant Factory with Artificial Lightning
LEDs	Light-Emitting Diodes
FW	Fresh Weight
GS	Glutamine Synthetase
GOGAT	Glutamate Synthase
MACl	Methoxyamine hydrochloride
